# Supplementary material for: HIVprotI: an integrated web based platform for prediction and design of HIV proteins inhibitors
Source: J Cheminform. 2018 Mar 9;10:12. doi: 10.1186/s13321-018-0266-y (PMC5845081; doi:10.1186/s13321-018-0266-y)
Supplement: Supplementary file 2 — Additional file 2. Source code of HIVProtI web server. [file 13321_2018_266_MOESM2_ESM.zip › HIVprotI_Source-code/hivprotI/weka-3-6-6/doc/weka/core/logging/Logger.Level.html]

Logger.Level


---


|  |  |  |
| --- | --- | --- |
| **Overview** \| **Package** \| **Class** \| **Tree** \| **Deprecated** \| **Index** \| **Help** \| **Weka's home** | |  |
| **PREV CLASS**   **NEXT CLASS** | **FRAMES**    **NO FRAMES**     **All Classes** |  |
| SUMMARY: NESTED \| ENUM CONSTANTS \| FIELD \| METHOD | DETAIL: ENUM CONSTANTS \| FIELD \| METHOD |  |


---


## weka.core.logging Enum Logger.Level

```
java.lang.Object
  java.lang.Enum<Logger.Level>
      weka.core.logging.Logger.Level
```

**All Implemented Interfaces:**: java.io.Serializable, java.lang.Comparable<Logger.Level>

**Enclosing class:**: Logger

---

``` public static enum Logger.Level extends java.lang.Enum<Logger.Level> ```

The logging level.

**Version:**
:   $Revision: 4716 $

**Author:**
:   fracpete (fracpete at waikato dot ac dot nz)

---

**Enum Constant Summary**

`ALL`
  
          logs all messages.

`FINE`
  
          FINER level.

`FINER`
  
          FINEST level.

`FINEST`
  
          FINEST level.

`INFO`
  
          FINE level.

`OFF`
  
          turns logging off.

`SEVERE`
  
          SEVERE level.

`WARNING`
  
          WARNING level.


| **Method Summary** | |
| --- | --- |
| `int` | `getOrder()`             Returns the order of this level. |
| `static Logger.Level` | `valueOf(java.lang.String name)`             Returns the enum constant of this type with the specified name. |
| `static Logger.Level[]` | `values()`             Returns an array containing the constants of this enum type, in the order they are declared. |

**Methods inherited from class java.lang.Enum**

`compareTo, equals, getDeclaringClass, hashCode, name, ordinal, toString, valueOf`

**Methods inherited from class java.lang.Object**

`getClass, notify, notifyAll, wait, wait, wait`

**Enum Constant Detail**

### ALL

```
public static final Logger.Level ALL
```

:   logs all messages.

---


### FINEST

```
public static final Logger.Level FINEST
```

:   FINEST level.

---


### FINER

```
public static final Logger.Level FINER
```

:   FINEST level.

---


### FINE

```
public static final Logger.Level FINE
```

:   FINER level.

---


### INFO

```
public static final Logger.Level INFO
```

:   FINE level.

---


### WARNING

```
public static final Logger.Level WARNING
```

:   WARNING level.

---


### SEVERE

```
public static final Logger.Level SEVERE
```

:   SEVERE level.

---


### OFF

```
public static final Logger.Level OFF
```

:   turns logging off.


**Method Detail**

### values

```
public static Logger.Level[] values()
```

:   Returns an array containing the constants of this enum type, in
    the order they are declared. This method may be used to iterate
    over the constants as follows:

    ```
    for (Logger.Level c : Logger.Level.values())
        System.out.println(c);
    ```

    :   **Returns:**: an array containing the constants of this enum type, in the order they are declared

---


### valueOf

```
public static Logger.Level valueOf(java.lang.String name)
```

:   Returns the enum constant of this type with the specified name.
    The string must match *exactly* an identifier used to declare an
    enum constant in this type. (Extraneous whitespace characters are
    not permitted.)

    :   **Parameters:**: `name` - the name of the enum constant to be returned. **Returns:**: the enum constant with the specified name **Throws:**: `java.lang.IllegalArgumentException` - if this enum type has no constant with the specified name: `java.lang.NullPointerException` - if the argument is null

---


### getOrder

```
public int getOrder()
```

:   Returns the order of this level.

    :   **Returns:**: the order


---


|  |  |  |
| --- | --- | --- |
| **Overview** \| **Package** \| **Class** \| **Tree** \| **Deprecated** \| **Index** \| **Help** \| **Weka's home** | |  |
| **PREV CLASS**   **NEXT CLASS** | **FRAMES**    **NO FRAMES**     **All Classes** |  |
| SUMMARY: NESTED \| ENUM CONSTANTS \| FIELD \| METHOD | DETAIL: ENUM CONSTANTS \| FIELD \| METHOD |  |


---
